# Merkel cell carcinoma in collision with squamous cell carcinoma and basal cell carcinoma in a patient on ruxolitinib treatment for myeloproliferative neoplasm

**DOI:** 10.1016/j.jdcr.2024.01.024

**Published:** 2024-02-02

**Authors:** Viviana Barrera-Penagos, Daniela Castellanos-Leguizamón, José Antonio Hakim-Tawil, Juan José Santivañez, Paula A. Rodríguez-Urrego, Juan Carlos Hiromi López-Takegami

**Affiliations:** a Department of Pathology and Laboratories, Fundación Santa Fe de Bogotá, Bogotá D.C., Colombia; b Division of Head and Neck Surgery, Fundación Santa Fe de Bogotá, Bogotá D.C., Colombia; c School of Medicine, Universidad de los Andes, Bogotá D.C., Colombia

**Keywords:** basal cell carcinoma, collision tumor, Janus kinase inhibitors, Merkel cell carcinoma, ruxolitinib, squamous cell carcinoma, synchronous tumor

## Introduction

Janus kinase inhibitors (JAK-I) have been gaining importance in the treatment of myeloproliferative neoplasms (MPNs), such as myelofibrosis and polycythemia vera.^1^ One of the most used JAK-I indicated for the treatment MPN is ruxolitinib, a potent oral selective inhibitor of JAK1 and JAK2.^1^ Its use has been shown to improve the quality of life of patients with MPN, reducing splenomegaly and symptom burden compared with the previous standard treatment.^2^^,^^3^ However, clinical trials that have evaluated the long-term effects of the use of ruxolitinib have found cases of newly diagnosed nonmelanoma skin cancer (NMSC) after 5 years of exposure among patients with myelofibrosis and polycythemia vera.^3^ Herein we present the case of a patient with a V617F JAK2-positive MPN treated with ruxolitinib, who developed multiple skin tumors after 6 years of treatment. Among these, 2 synchronous, fast-growing lesions emerged, composed of Merkel cell carcinoma (MCC) in collision with squamous cell carcinoma (SCC) and basal cell carcinoma (BCC). This case highlights the importance of active dermatological monitoring, especially in patients with sun skin-damage history and in treatment with JAK-I for myeloproliferative conditions.

## Case report

We present the case of an 80-year-old man diagnosed in 2012 with MPN positive for V617F JAK2 mutation. The patient received treatment with hydroxyurea from 2012 to 2015; however, it was changed because of intolerance to 20 mg of ruxolitinib twice a day. He remained stable and without any side effects for the following 2 years. The patient had a history of actinic keratosis related to sun exposure without any history of skin cancer until 2017, when he presented for the first time with multiple suspicious skin lesions. Within a 2-year timeframe, the patient was diagnosed with 2 BCCs affecting the lip and right postauricular region, along with 2 SCCs on the forehead and the lower right malar region. The patient underwent successful tumor removal for all cases. In addition, several clinically evident hyperkeratotic actinic keratoses were addressed through direct treatment with an electrosurgical scalpel. Treatment with ruxolitinib continued.

**Fig 1:**
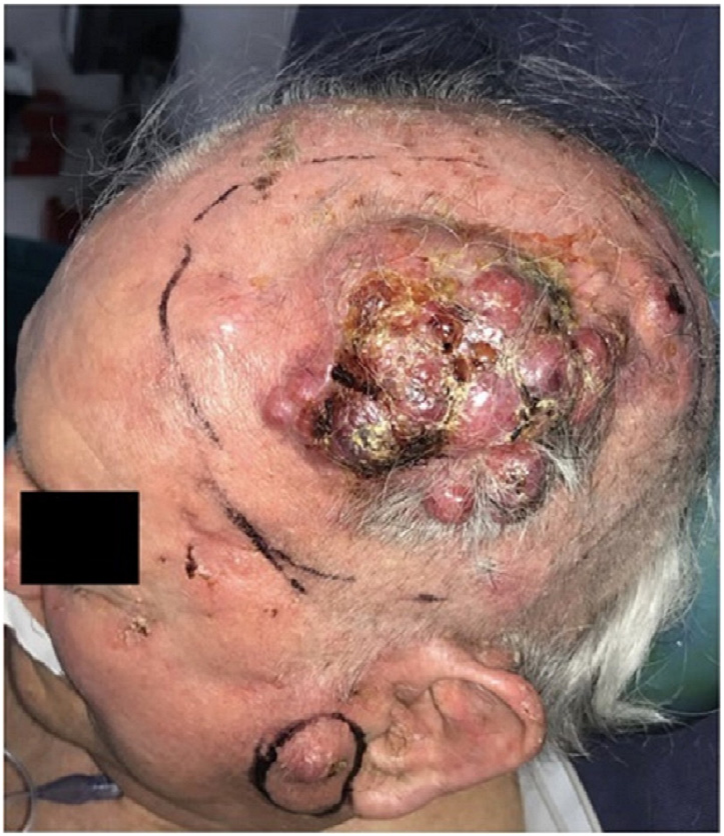
Clinical presentation. Left frontoparietal lesion presents as a multinodular and poor circumscribed ulcerated tumor. Left preauricular lesion presents as an indurated and erythematous nodule.

**Fig 2:**
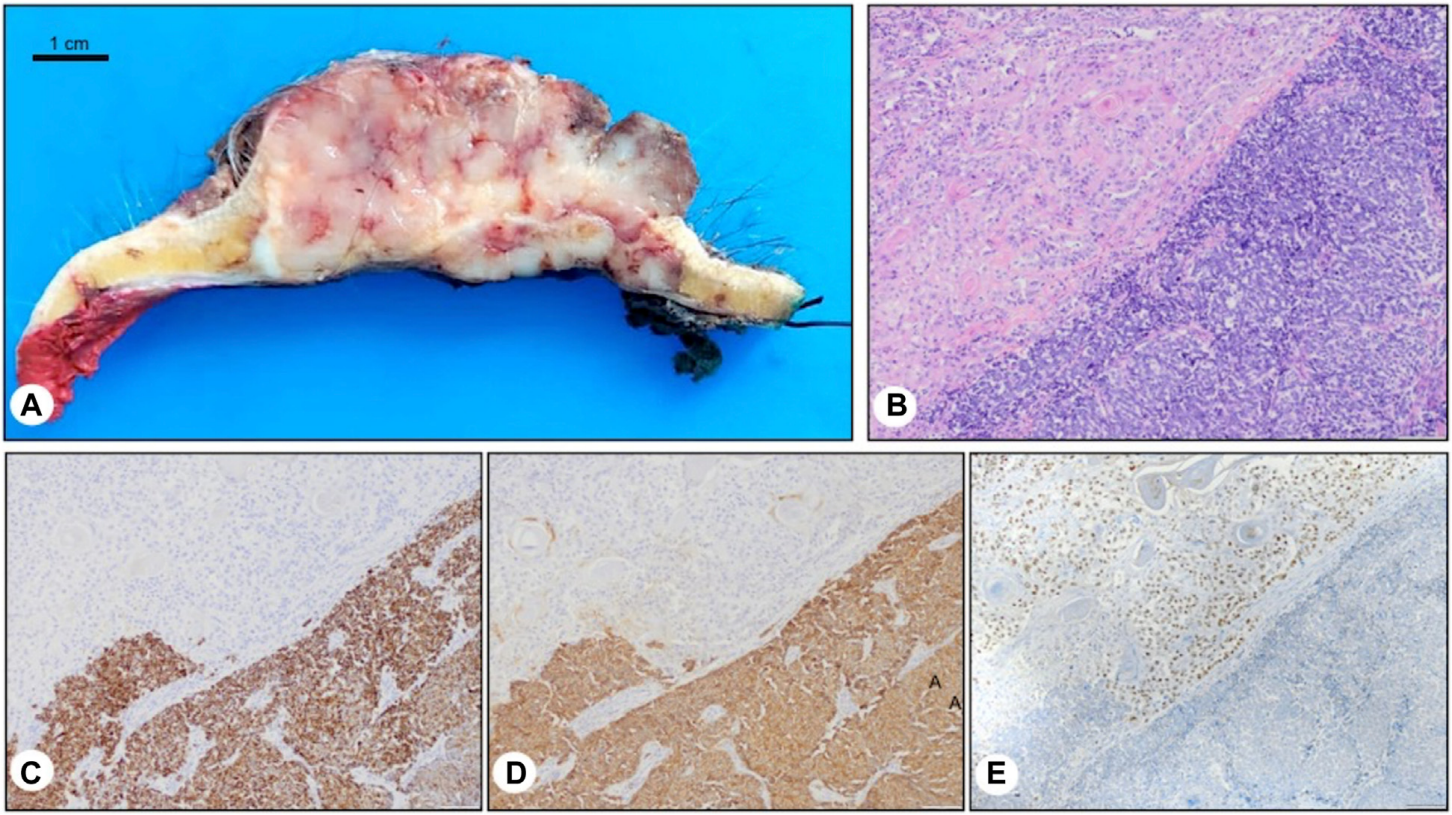
Scalp lesion, Merkel cell carcinoma (MCC) in collision with squamous cell carcinoma (SCC). **A,** Macroscopic findings, sagittal cut. **B,** Invasive, well-differentiated SCC next to MCC. **C,** CK20 reactive in MCC. **D,** Synaptophysin reactive in MCC. **E,** P40 reactive in SCC. (**B,** Hematoxylin-eosin stain; original magnifications: **B,** ×10; **C,** ×10; **D,** ×10; **E,** ×10.)

In 2021, the patient presented once more with rapidly growing, bleeding lesions located the left frontoparietal and left preauricular regions (Fig 1). Surgical resection was performed on both lesions, each undergoing a separate surgical procedure to ensure proper removal. The left frontoparietal lesion showed an ulcerated tumor measuring 7 × 7 × 2.5 cm, with macroscopic extension to the subcutaneous tissue. The microscopic study revealed a combination of 2 collision tumors. The dominant one showed sheets of small round blue cells with scant cytoplasm, salt and pepper chromatin, and lymphovascular invasion. Immunohistochemical staining showed CK20 cells with perinuclear dot staining, reactivity for synaptophysin and 95% KI67 stained cells. These findings were consistent with MCC. Testing for Merkel cell polyomavirus was not performed. The secondary tumor showed large, atypical squamous epithelium, abundant keratinization, apparent intercellular bridges, some nuclear pleomorphism, frequent mitotic figures, and perineural invasion, corresponding to an invasive, well-differentiated SCC (Fig 2).

**Fig 3:**
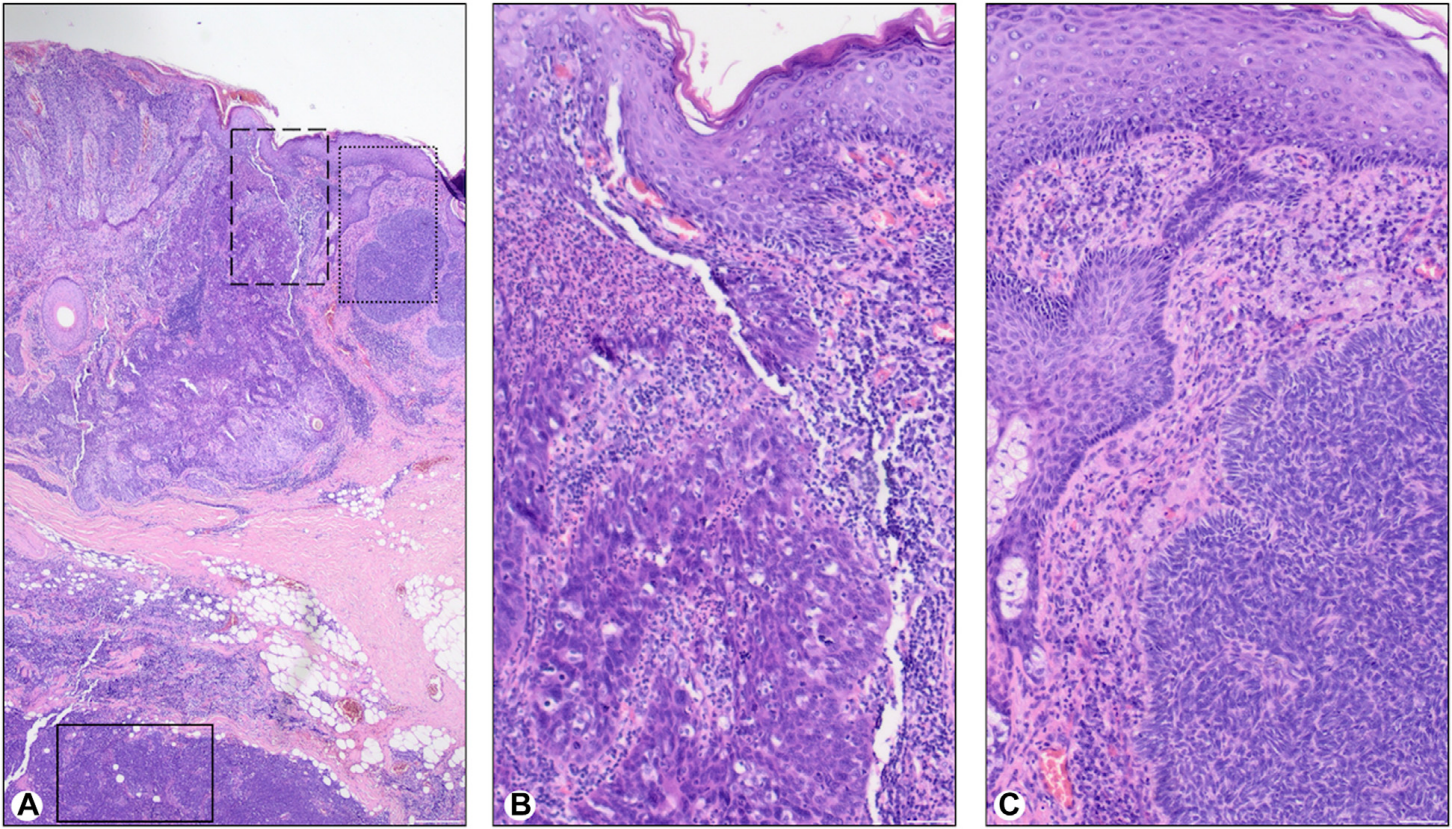
Preauricular lesion, Merkel cell carcinoma (MCC) in collision with squamous cell carcinoma (SCC) and nodular basal cell carcinoma (BCC). **A,** Panoramic view of the 3 tumors. The solid line box corresponds to MCC. **B,** SCC, a higher power of dashed line box. **C,** BCC, a higher power of dotted line box. (**A-C,** Hematoxylin-eosin stain; original magnifications: **A,** ×2; **B,** ×10; **C,** ×10.)

The specimen obtained from the left preauricular region showed an elevated 1.3 × 1.3 × 1.2–cm nodule. Microscopically, the tumor was composed of 3 different neoplasms: a dominant nodular MCC that showed the same findings described for the scalp lesion; and 2 secondary tumors with reticular dermis infiltration comprised of an invasive, well-differentiated SCC with atypical keratinocytes, loss of cell polarity and keratinization; and a nodular BCC with atypical basaloid keratinocytes with peripheral palisading (Fig 3).

After surgical removal of the lesions, hematooncology suspended ruxolitinib. The patient refused other additional therapies. He is being treated with warfarin and is undergoing periodical platelet-count follow-ups and continuous routine follow-ups with oncology. After the diagnosis of MCC, a PET-CT was performed, showing no signs of malignancy. Despite the discontinuation of ruxolitinib, the patient developed 3 new SCCs on the left temporal region and right frontal region between 2022 and 2023, all of which were surgically excised.

## Discussion

Prolonged JAK-I therapy has been related to the possible development of aggressive NMSC, with growing reported cases.^2^^,^^4^, ^5^, ^6^ The inhibition of the JAK-STAT pathway by JAK-I has shown satisfactory results against some types of malignancies; however, the impairment of this pathway also weakens pro-inflammatory interleukin and interferon secretion, thus potentially weakening the cytotoxic-T-cell activation and function, and resulting in an enhanced environment for malignant cell development.^4^ Skin cancer, particularly MCC, has been related to the sun-exposed skin of patients.^2^^,^^7^ MCC incidence is higher in patients with HIV/AIDS, hematological diseases, and immunosuppression.^2^ MCC often presents in association with other cutaneous neoplasms within the same lesion, including trichoblastoma, BCC or SCC.^8^ As reported, the most prevalent association is with invasive or in situ SCC, accounting for 5% to 35% of all MCC cases.^8^ In our case, the presence of 2 MCCs may suggest that one of them is a metastasis, taking into account the high metastasis rate of these tumors.^9^ However, in rare cases, a second primary MCC may arise, in which case confirmation is done through molecular studies.^9^ The collision between MCC with SCC and BCC, observed in both lesions developed by our patient, might favor a case of a second primary MCC, as suggested in previous reports.^9^ Unfortunately, we were not able to perform molecular studies to confirm this hypothesis. In addition, patients who have been diagnosed with actinic keratosis show a higher risk of developing NMSC and malignant melanoma in the 10 years following diagnosis.^7^ Thus, previous skin cell damage due to ultra violet light in addition to the immunosuppression derived from the treatment for MPN far increases the development of skin cancer.

This case shows the progression of 3 collision tumors distributed in 2 synchronous lesions, with a rapid onset and growth facilitated by previous sun cell damage and current immunosuppression. The history of rapidly growing and progressively more aggressive cancerous lesions in this patient after 2 years of starting immunosuppressive therapy, without any other relevant history of skin cancer, suggests that the use of ruxolitinib influenced the development of these neoplasms. As a result, it is wise to consider the reported association between the use of JAK-I and the development of NMSC when providing treatment for MPN. In hopes of reducing the incidence and/or facilitating a prompt diagnosis of skin cancer, referring patients for periodical dermatology checks after treatment initiation with ruxolitinib is highly recommended.^10^ Specifically, we advise skin screening 2 to 3 times a year for all patients under treatment. Even in the absence of a history of NMSC, if skin tumors develop during or after JAK-I therapy, we recommend more frequent dermatological monitoring, up to 4 times a year.

## Conflicts of interest

None disclosed.
